# Grouting technique—combining transvenous Onyx and coils for the embolization of complex non-cavernous intracranial dural arteriovenous fistulas

**DOI:** 10.3389/fneur.2024.1416945

**Published:** 2024-07-22

**Authors:** Ching-Chang Chen, Chun-Ting Chen, Yi-Ming Wu, Mun-Chun Yeap, Yao-Liang Chen, Ho-Fai Wong

**Affiliations:** ^1^Department of Neurosurgery, Chang Gung Memorial Hospital, Linkou, Chang Gung University and Medical College, Taoyuan, Taiwan; ^2^Department of Radiology, Chang Gung Memorial Hospital, Linkou, Chang Gung University and Medical College, Taoyuan, Taiwan

**Keywords:** dural arteriovenous fistula, endovascular, transvenous approach, coil embolization, Onyx

## Abstract

**Objective:**

Incomplete occlusion of cerebral dural arteriovenous fistula (DAVF) may lead to fistula recurrence and rebleeding, which may necessitate several embolizations and lead to worse clinical outcomes. Herein, we describe a grouting technique for endovascular embolization and its outcomes in a series of patients with complex intracranial DAVF.

**Methods:**

A total of 20 patients with aggressive type or symptomatic intracranial non-cavernous DAVF underwent endovascular transvenous embolization combining detachable coils and Onyx. Two microcatheters were positioned either in the distal segment of the involved sinus or near the draining veins. To achieve tight occlusion of the involved sinus, coils were carefully delivered through the first microcatheter, starting from the distal segment and then to the proximal segment. Next, Onyx was injected through the second microcatheter to reinforce and fill (grout) the interspace of coil mass and gradually refluxed to the mural channels and para-sinus cortical veins until the fistula was completely occluded.

**Results:**

Successful embolization was achieved in all 20 patients. The initial angiographic results revealed the achievement of complete occlusion in 19 patients (95%). At the postembolization follow-up, complete obliteration of the fistula was achieved in all patients (100%). No symptom or angiographic recurrence was observed at the 2- to 5-year follow-ups. No patient required additional embolization or stereotactic radiosurgery.

**Conclusion:**

The proposed grouting technique combining detachable coils and Onyx appears to be promising for the elimination of complex intracranial non-cavernous DAVFs.

## 1 Introduction

Endovascular therapy is currently the first-line treatment for most cases of ruptured or high-grade dual arteriovenous fistulas (DAVFs). Transvenous obliteration is an effective approach for directly reducing the size of the venous collector and the reflux into the cortical veins (1–3). In cases where the involved sinus segment contributes minimally to the normal cerebral outflow, transvenous packing with detachable coils can be a relatively safe approach for achieving complete occlusion (1–3). However, tight packing and the complete elimination of the involved sinus must be ensured. Otherwise, the high pressure may be diverted toward the cortical venous system and result in cerebral hemorrhage and venous infarction (4–6). Achieving complete obliteration using coils alone may be challenging in patients with multiple intra-sinus septa, trabecula, and complex parallel venous channels (7, 8) that communicate with, but are distinct from, the sinus. Incomplete occlusion of DAVFs may lead to fistula recurrence and rebleeding, necessitating several embolizations and compromising clinical outcomes (8). Additional Onyx embolic material may be effective when used in a transarterial approach or combined with coils. Such an approach has been demonstrated in some case series on cavernous sinus fistula (1, 5, 9). In this study, we describe a one-stage endovascular grouting technique that involves filling a scaffolded coil mass with Onyx to achieve the complete and effective occlusion of complex intracranial non-cavernous DAVFs.

## 2 Methods

### 2.1 Study population and clinical presentation

We retrospectively analyzed the data of 112 consecutive patients who presented to our hospital with intracranial DAVF between January 2015 and December 2022. All patients opted for endovascular management, and 20 patients agreed to undergo embolization following the proposed grouting technique. The patients’ initial clinical presentations were evaluated. All intracranial conditions, including intracranial hemorrhage, sinus thrombosis or occlusion, and venous infarction, were validated through computed tomography (CT) or magnetic resonance imaging (MRI). Before treatment, angioarchitectural explorations were performed through formal diagnostic subtraction angiography (DSA) to assess fistulous sites, feeding arteries, sinus thrombosis or occlusion, and venous drainage. The contributions of the involved sinus segment to the normal cerebral outflow were analyzed. Cognard classification was used to identify cortical venous reflux. General patient characteristics are summarized in Table 1.

**Table 1:** Baseline characteristics of patients with dural arteriovenous fistulas who underwent embolization performed using the proposed grouting technique.

Case	Age + (decade)	Presentation	Location	Type	Coil^a^/Onyx (in mL)	Complications	Angiographic results	Recur	Follow-up period (in years)
1	70+	Tinnitus	Right TS	IIa + b	20/1	No	Complete	No	2
2	50+	ICH	Left TS and SS	III	25/1.5	No	Complete	No	2
3	50+	ICH	Left TS and SS	IIa + b	25/2	No	Complete	No	2
4	60+	ICH	SSS	IIa + b	22/2	No	Nearly complete	No	3
5	50+	ICH	Right TS and SS	IIa + b	15/1	No	Complete	No	3
6	50+	Tinnitus	Right TS and SS	IIa	20/0.5	No	Complete	No	3
7	60+	Tinnitus	Right TS	IIa + b	20/1	No	Complete	No	3
8	30+	Tinnitus	Left TS and SS	IIa	18/0.5	No	Complete	No	3
9	60+	ICH and seizure	Right TS, SSS, and torcula	IIa + b	20/1.3	No	Complete	No	3
10	70+	Tinnitus	Left TS	IIb	20/1.5	No	Complete	No	5
11	50+	Tinnitus	Left TS	IIa + b	15/1	No	Complete	No	3
12	60+	ICH and IVH	Vein of Galen	IV	25/1.5	No	Complete	No	5
13	60+	Tinnitus	Right TS and SS	IIa	20/1	No	Complete	No	5
14	50+	Tinnitus and facial palsy	Left CS and left hypoglossal	IIa + b	23/1.2	No	Complete	No	5
15	60+	ICH	Right TS	IIa + b	11/0.8	No	Complete	No	3
16	60+	Tinnitus	Left TS and SS	IIa	15/1	No	Complete	No	3
17	50+	Tinnitus	Left SS	IIa + b	18/0.5	No	Complete	No	3
18	50+	Syncope and tinnitus	Right TS and SS	IIa + b	10/0.3	No	Complete	No	2
19	70+	ICH	Left torcula and TS	III	20/1	No	Complete	No	2
20	50+	Tinnitus	Right SS	IIa + b	20/1	No	Complete	No	2

M, male; F, female; TS, transverse sinus; SS, sigmoid sinus; SSS, superior sagittal sinus; CS, cavernous sinus; ICH, intracranial hemorrhage; IVH, intraventricular hemorrhage.

a Number of coils.

In our center, If the involved sinus can be preserved, we will give first priority to preserve the sinus through transarterial liquid embolizer combined with balloon or stent to protection in the sinus. Compete obliteration could also be reached possibly through simply transarterial approach or pressure cooker technique if the DAVF was supplied by a single feeder. DAVFs developed in frontal ethmoidal sinus or foramen magnum would be treated by direct surgical devascularization first. We would not intentionally use the technique in cavernous sinus to prevent further cranial palsy. Therefore, the grouting technique was only applied for sinus sacrifice when the involved sinus segment contributes nothing or minimally to the normal outflow.

Therapeutic modalities were discussed with both the neurosurgical and neurointerventional teams. After receiving consultation, all patients provided informed consent. This study was approved by our institute’s institutional review board (IRB: 202300612B0). The requirement for written informed consent was waived.

### 2.2 Embolization procedure

Endovascular procedures were performed with the patient under general anesthesia; transarterial and transvenous punctures were performed through the femoral artery and vein for road mapping and embolization, respectively. First, we introduced a 5-Fr diagnostic catheter through the external carotid artery into the main feeding arteries and then navigated a 6-Fr guiding catheter into the main draining (i.e., jugular) veins. The two 1.7-Fr microcatheters were placed in the distal segment of the involved sinus or the nearest draining veins. The angioanatomic configurations of the DAVF were defined through selective angiography. Thereafter, embolization was performed. One microcatheter was used to deliver detachable coils to achieve tight occlusion of the involved sinus or the draining veins in the distal-to-proximal direction. When complete fistula occlusion could not be achieved using coils, a liquid material (Onyx-18; Ev3 Neurovascular, Irvine, CA, United States) was injected through the second microcatheter, which was still buried in the sinus. During injection, the distribution of Onyx was monitored through continuous fluoroscopy. In most cases, Onyx was contained by the coil mass and the remaining small residual sinus compartments were filled with Onyx, allowing it to gradually reflux into the mural channels of the sinus and the associated cortical drainage veins. The procedure was similar to grout concrete into the steel framework or fill the gaps of the bricks when building construction. Onyx was injected to reinforce and fill the interspace of coil mass and involved sinus. Four-vessel DSA was performed immediately after embolization. Most of the patients were extubated in the angiographic room and discharged 24–48 h after the surgery. For patients with preoperative neurologic deficits, further rehabilitation could be arranged within few days.

Clinical outcomes were evaluated on the basis of postoperative complications, such as hemorrhagic or ischemic stroke or a new neurological or ocular deficit. According to the degree of shunting, angiography results were classified as follows: complete occlusion (i.e., no shunt), near-complete occlusion (i.e., minor residual shunt with considerable volume and velocity reductions), and partial occlusion (major residual shunt with little to no reduction in volume and velocity). If the fistula was not completely occluded immediately after treatment, DSA was arranged within 6 months or once the symptoms early recurred. If all fistulas were completely occluded, clinical follow-up and imaging studies (DSA) were advised 1 year after treatment. Thereafter, clinical and magnetic resonance angiography follow-up continued annually in the outpatient department.

## 3 Result

### 3.1 Study population and presentation

This retrospective study included the 20 patients who underwent embolization performed using the grouting technique. Data regarding the patients’ baseline characteristics, initial main presentations, fistula type (Cognard classification), and treatment outcomes are summarized in Table 1. Eleven men and nine women were evaluated, and the mean age was 59.35 years. The fistulas of 17 patients (85%) involved the transverse, sigmoid sinus or both. One patient had a fistula in the superior sagittal sinus, one in the vein of Galen, and one in the hypoglossal sinus. Aggressive symptoms such as cerebral hemorrhage or ischemia stroke developed in nine patients (45%). The initial angiographic findings revealed cortical venous reflux (Cognard classes IIb, IIa + b, III, and IV) in 16 fistulas (80%). Patients with lower classification (20%) were treatment due to bothering tinnitus.

### 3.2 Endovascular and clinical outcomes

Embolization was successful for all 20 patients. According to the initial angiographic results, complete occlusion was achieved in 19 (95%) patients. During postembolization follow-up, complete obliteration of the fistula was achieved in all 20 patients (100%). No symptom or angiographic recurrence was observed at the 2- to 5-year follow-ups. Therefore, no patient required additional embolization or stereotactic radiosurgery.

### 3.3 Case demonstration

#### 3.3.1 Case 1

**Figure 1:**
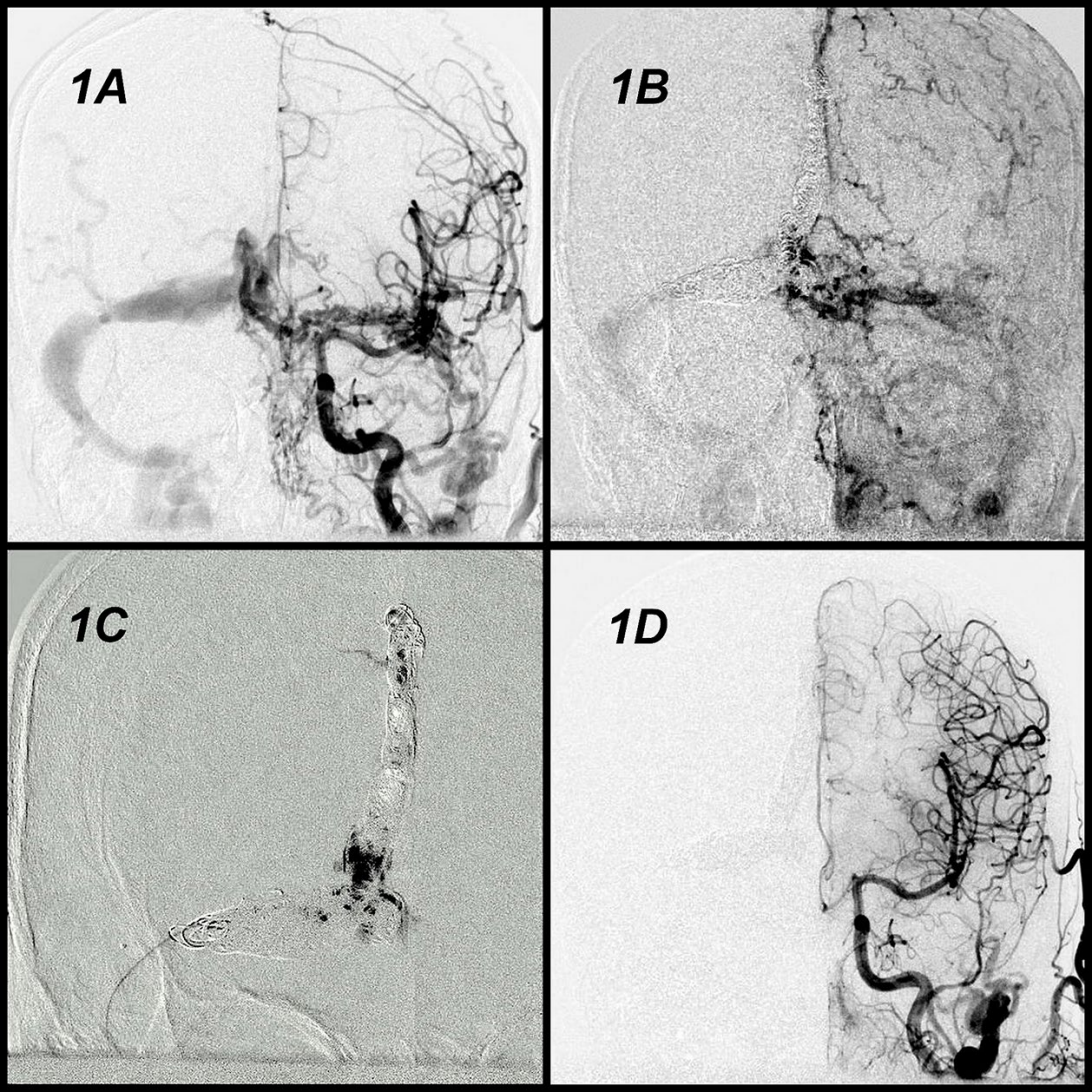
Angiographic findings and endovascular results obtained in Case 1. A posterior fossa DAVF located at the junction of the posterior third of the superior sagittal sinus, torculas, and right transverse sinus. The DAVF was classified as Cognard type IIa + b with abnormal blood supplies **(A)**. Coil embolization of the entire involved sinus **(B)**. Onyx was injected to fill the interspace of the coil mass **(C)**. The fistula was completely occluded **(D)**. DAVF, dural arteriovenous fistula.

A man in the early sixties (Case 4 in Table 1) presented to our hospital with sudden-onset syncope and seizure. CT-angiography revealed a mild subarachnoid hemorrhage with multiple twisted vessels in the right frontal and temporal regions of the brain. The angiography also revealed DAVF in the right posterior fossa; the fistula was located at the junction of the posterior third of the superior sagittal sinus, torculas, and right transverse sinus. The DAVF was classified as Cognard type IIa + b and had complicated blood supplies originating from the bilateral occipital arteries, middle meningeal arteries, and tentorial branch of the meningohypophyseal trunk (Figure 1A). After exhaustive review of the angioarchitecture, a venous approach was adopted to eliminate a part of the involved sinus. First, coil embolization of the entire involved sinus (Figure 1B) was performed. Although cortical venous reflux ceased and the flow decreased, shunting persisted. The procedure could be terminated here, and the patient was evaluated at clinical and imaging follow-ups; or the procedure could continue by exchanging the approach route to transarterial approach to reduce arterial flow. Rather than doing these, we used the preburied second microcatheter and injected Onyx to fill the coil mass interspace (Figure 1C) and nearly completely occluded the DAVF (Figure 1D). Complete occlusion was reached without recurrence of the lesion was observed at the 1-year angiographic follow-up, and the patient was seizure-free at the 3-year clinical follow-up.

#### 3.3.2 Case 2

**Figure 2:**
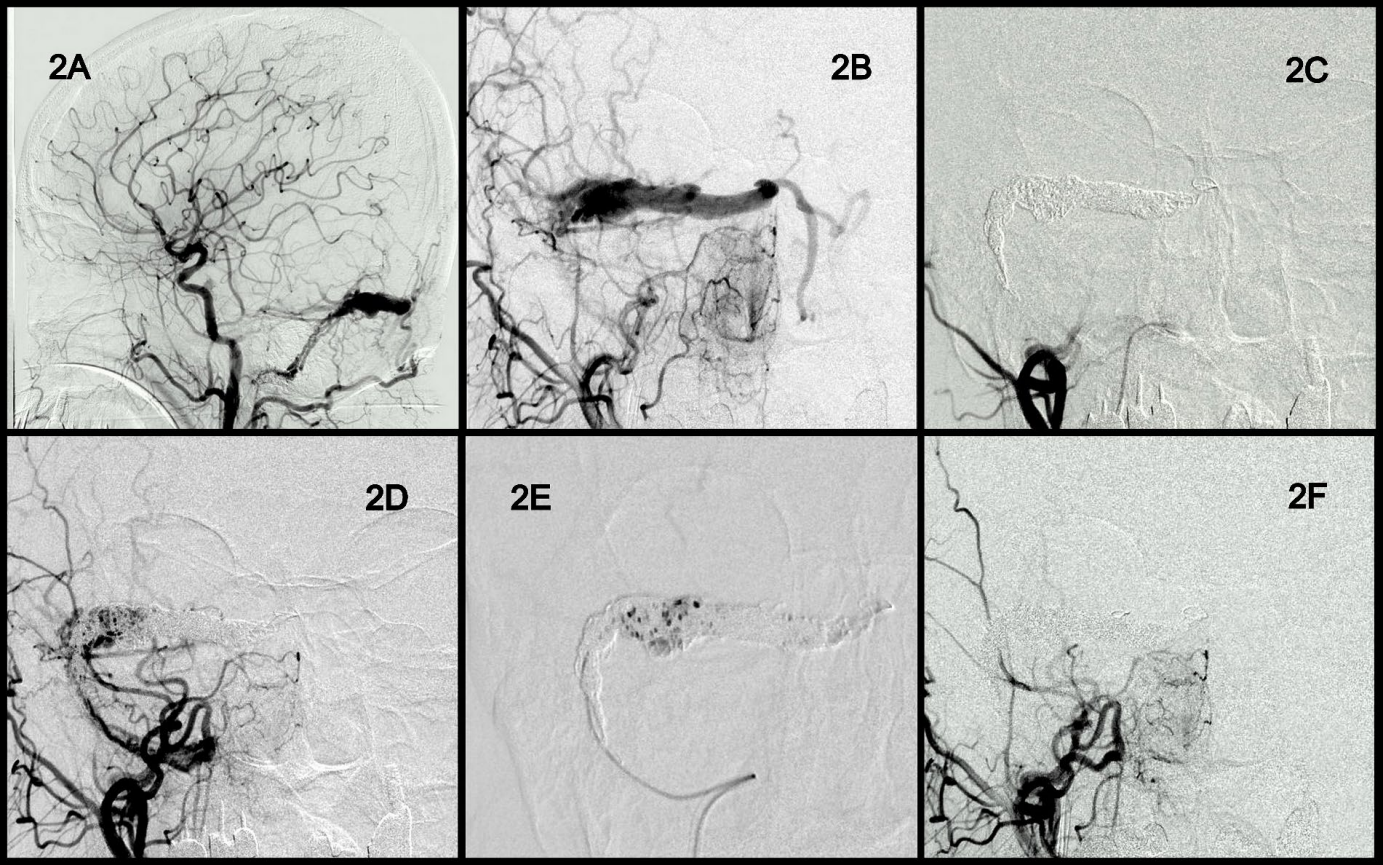
Angiographic findings and endovascular results obtained in Case 2. Anterior and lateral view of the right transverse and sigmoid sinus DAVF with multiple cortical venous reflux. The DAVF was classified as Cognard type IIa + b **(A,B)**. Coils were delivered for the embolization of the entire involved sinus **(C)**. Residual sinus filling and venous reflux **(D)**. Onyx injection from the second microcatheter **(E)**. Complete occlusion of the DAVF **(F)**. DAVF, dural arteriovenous fistula.

A woman in the fifties (Case 3 in Table 1) presented to our hospital with progressive headache and drowsiness. Brain CT revealed a small hemorrhage and severe brain edema. Angiography showed a right transverse and sigmoid sinus DAVF with multiple cortical venous refluxes. The DAVF was classified as Cognard type IIa + b (Figures 2A,B). Coils were delivered from the first microcatheter to embolize the entire involved sinus (Figure 2C). However, some residual sinus filling and venous reflux remained (Figure 2D). Onyx was injected from the second microcatheter (Figure 2E), and complete occlusion of the DAVF was achieved (Figure 2F). The patient gradually regained consciousness, and no recurrence of the fistula was observed at the 1-year angiographic follow-up.

#### 3.3.3 Case 3

**Figure 3:**
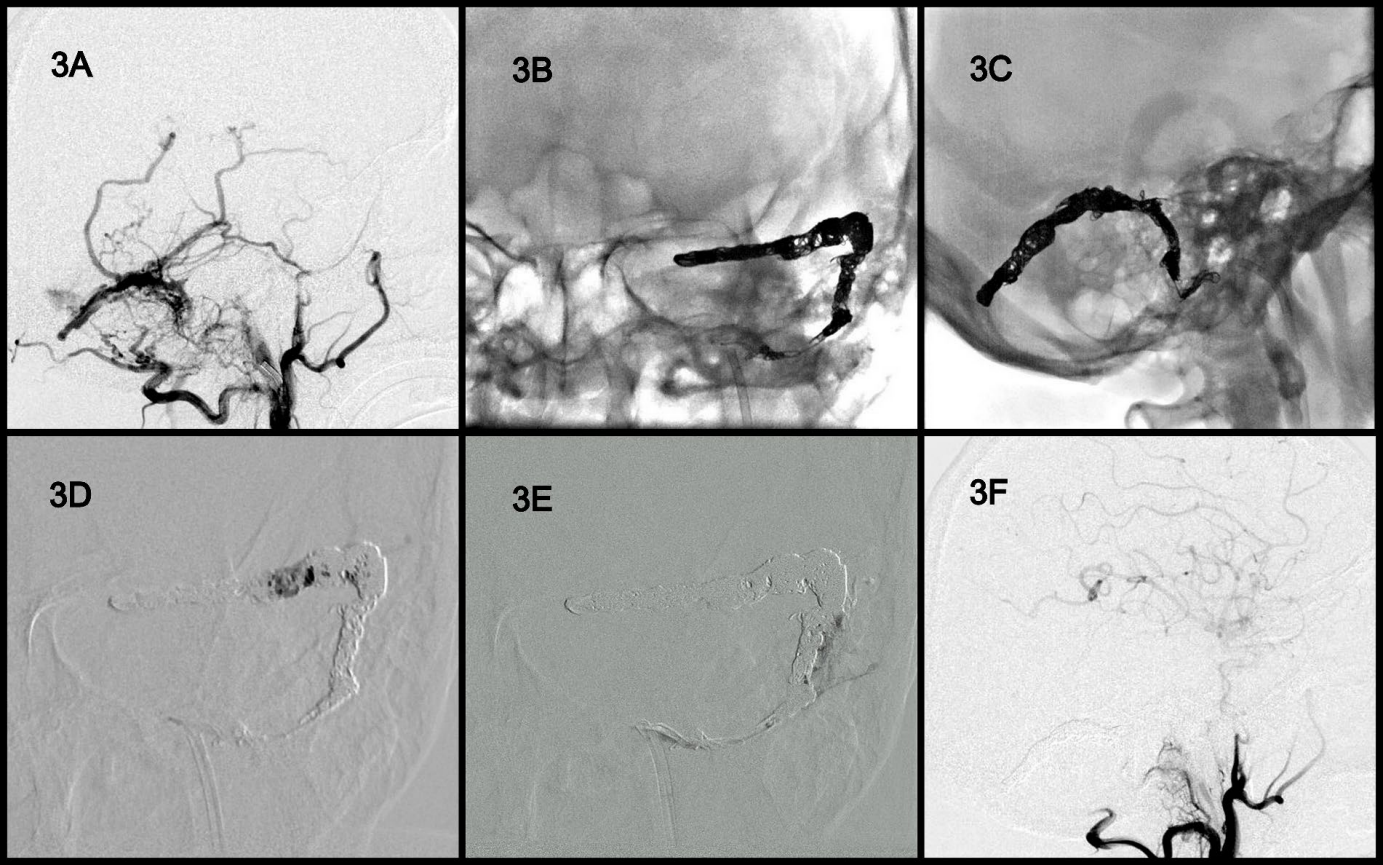
Angiographic findings obtained in Case 3. DAVFs were located at the left transverse and sigmoid sinuses and the jugular bulb and were classified as Cognard 4 **(A)**. Coils were first delivered to the involved sinus **(B,C)**. Onyx was poured into the coil mass from the distal segment **(D)** to the proximal segment **(E)** to fill the residual sinus compartments and parallel venous channels. Complete obliteration of the fistula **(F)**. DAVF, dural arteriovenous fistula.

A man in the mid-fifties (Case 2 in Table 1) experienced pulsatile tinnitus for months and was finally referred to our institute by a local ENT specialist. Small old ICH was noted in brain MRI and angiography revealed the presence of DAVFs at the left transverse and sigmoid sinuses and the jugular bulb. The DAVFs were classified as Cognard III (Figure 3A). Similar to the aforementioned case, the coils were first delivered to embolize the involved sinus (Figures 3B,C). Next, Onyx was poured into the coil-mass scaffold and withdrawn slowly from the distal segment (Figure 3D) and then the proximal segment (Figure 3E). Consequently, the residual sinus compartments and parallel venous channels that were near or outside the sinus wall were filled with Onyx (Figure 3E). Complete obliteration was achieved (Figure 3F). The patient’s tinnitus disappeared immediately after the procedure without any discomfort or complications.

#### 3.3.4 Case 4

**Figure 4:**
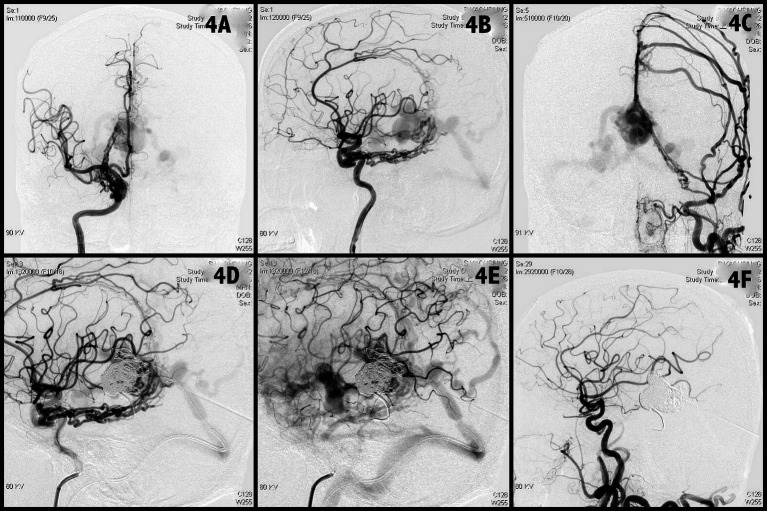
Angiographic findings obtained in Case 4. A high flow vein of Galen DAVF with multiple feeders from bilateral ICAs, ECAs, VAs with drainage to straight sinus, superficial, and deep cortical veins was noted **(A–C)**. Transvenous dense coils packing of the vein of Galen **(D)**. But still persistent high flow cortical reflux was found **(E)**. The grouting technique was used to fill the residual sinus interspace and mural channels gradually. The entire fistula was completely occluded **(F)**. DAVF, dural arteriovenous fistula; ICA, internal carotid artery; ECA, external carotid artery; VA, vertebral artery.

A man in the early thirties (Case 12 in Table 1) experienced conscious change due to intracranial hemorrhage (ICH) and intra-ventricular hemorrhage (IVH). After ventricular drainage surgery, his conscious regained. Angiography revealed a high flow AV shunting to Vein of Galen, resulting aneurysmal dilatation of Vein of Galen and recruitment of venous drainage to deep and superficial veins. Numerous feeding arteries contribute the fistula include marginal arteries from bilateral ICAs (Figures 4A,B), middle meningeal arteries, occipital arteries of bilateral ECAs (Figure 4C), and choroidal meningeal artery from left PCA. Two microcatheters were navigated into the aneurysmal dilatation sac of Vein of Galen from right venous access. The aneurysmal sac was embolized with detachable coils as dense as possible (Figure 4D). However, still persistent high flow shunting with cortical reflux was found (Figure 4E). The grouting technique (Onyx) was used to fill the residual sinus interspace and mural channels on the sinus wall gradually. The entire fistula was completely occluded (Figure 4F) after deployed 25 coils and 1.5 mL Onyx. The patient’s condition improved and accepted surgery of ventriculoperitoneal shunt 1 week later. No recurrence of symptoms or the fistula was observed at the 5-year clinical follow-up and 2-year imaging follow up.

#### 3.3.5 Case 5

**Figure 5:**
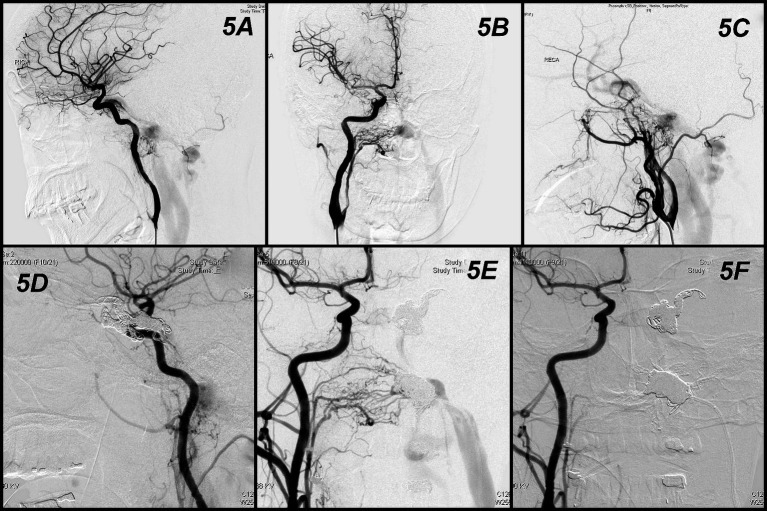
Angiographic findings and results obtained in Case 5. Two DAVFs **(A)**. One at left hypoglossal canal supplied from bilateral ascending pharyngeal arteries and occipital arteries and drained to internal jugular vein and inferior petrosal sinus **(B,C)**. Another left cavernous sinus DAVF supplied from bilateral ICAs and MMAs with retrograde flow to middle cerebral vein and superior ophthalmic vein **(A,C)**. Compete embolization of the left cavernous sinus first by detachable coils **(D)**. Persistent flow of fistula despite dense packing by coils in the hypoglossal canal **(E)**. The Onyx was used to completely occlude the fistula **(F)**.

A female in the mid-forties (Case 14 in Table 1) suffered from headache, pulsatile tinnitus, and left facial palsy. Angiography found two DAVFs (Figure 5A). One left hypoglossal canal AVF, which was supplied from bilateral ascending pharyngeal arteries and occipital arteries; drained to internal jugular vein and inferior petrosal sinus (Figures 5B,C). Another left cavernous sinus DAVF was supplied from bilateral ICAs and MMAs with retrograde flow to middle cerebral vein and superior ophthalmic vein (Figures 5A,C). Compete embolization of the left cavernous sinus first by detachable coils (Figure 5D). Microcatheter later shifted to hypoglossal canal and did coils embolization. However, persistent flow of fistula despite dense packing by 9 coils in the hypoglossal canal (Figure 5E). The 1.2 mL Onyx was used to completely occlude the fistula (Figure 5F). The patient’s tinnitus was ceased immediately after procedure and facial palsy gradually improved within 3 months.

## 4 Discussion

In this study, we analyzed patients with DAVF who underwent grouting technique (endovascular treatment with coils framework and following filling with Onyx injection) through one session of transvenous access. After long-term follow-up, a high rate of complete DAVF occlusion was observed without any increase in postoperative complications. Studies have consistently revealed that transarterial embolization often requires liquid agents, whereas transvenous embolization typically requires coils. Both techniques require multiple sessions of embolizations for the complete occlusion of fistula (8, 10). Some scattered case report or small studies reporting similar grouting technique with coils and liquid material only in the cavernous sinus. In spite of not the first report, the series is the largest and first demonstrated in non-cavernous sinus with long-term follow-up.

### 4.1 Transvenous approach and complete DAVF occlusion in a single session

For our patients, an endovascular approach was adopted as the first-line strategy. All patients underwent transvenous embolization. Transvenous obliteration is generally considered to be more effective than the transarterial approach, particularly in cases where the venous collector can be eliminated. The cure rate for this method ranges from 80–100% (1, 3, 6, 11, 12). Compete obliteration was sometimes not easy to reach through simply transarterial approach unless the DAVF was supplied by a single feeder. Otherwise, transarterial approach was often needed to combine pressure cooker technique or balloon/stent protection in the sinus (13, 14). Recently, by the development and maturity of pressure cooker technique, more and more fistula could be done by transarterial route. However, if the complete occlusion could not be guaranteed or the solitary sinus had no function but multiple feeders and reflux, transvenous approach was usually the one of choices (11). When the involved sinus segment contributes minimally to the normal outflow, the transvenous approach is considered to be relatively safe and allows for complete occlusion (6). Transvenous elimination of one of two paired dural sinuses, such as the sigmoid or transverse, may be acceptable as long as the contralateral channel is left intact. Most dural sinuses, including large segments of the superior sagittal sinus, can be obliterated safely (12).

Most DAVFs result from complex microscopic fistulas within the wall of the thrombosed or involved venous sinus, which connects the vasa vasorum to small venous tributaries and cause reflux into the cortical veins. When the arterial feeders converge on multiple parallel venous channels that communicate with, but are distinct from, the sinus, the obliteration of this channel results in the elimination of the fistula (7). Transvenous packing of the involved venous collector or dural sinus is most commonly performed using detachable coils. However, dense packing must be performed to prevent residual fistula and subsequent cortical venous drainage. Achieving complete occlusion through coils alone can be challenging because of factors such as multiple separated chambers, intra-sinus septa, and small parallel venous channels located near or outside the sinus wall (15). Therefore, multiple sessions of embolizations using the adjuvant transarterial approach are necessary to address DAVF recurrence. Furthermore, incomplete closure of the arteriovenous fistula shunt after the occlusion of the involved sinus may lead to the redirection of high-pressure shunting flow toward the cortical vein, which increases the risk of hemorrhage (1, 4, 6, 7).

A double-microcatheter technique can increase the density of packed coils (1, 2). If complete occlusion of DAVFs cannot be achieved using coils alone, a liquid injection may be necessary. The injection is administered through the microcatheter positioned inside the sinus for the immediate and complete occlusion of the fistula (5, 16). Consequently, the liquid embolic material gradually and conveniently reaches the cortical vein exhibiting reflux, inter-sinus connections, and other venous outlets in the sinus wall. This method was achieved using a liquid embolic material that easily diffused into areas where the coil mass alone could not facilitate effective occlusion (5, 16, 17). However, the coil mass may prevent the liquid material from excessively penetrating or rapidly flowing (in an antegrade direction) into uninvolved sinuses or even the heart. In this study, we performed the complete occlusion of DVAFs in a single session. Comparing our results with those of other studies is difficult because in most studies have reported a small series of cases or have included mixed groups of patients with DAVF of the cavernous sinus (1, 5, 15, 16, 18, 19). The rate of DAVF occlusion (as noted on angiography) was 95% immediately after embolization and 100% at the 1-year follow-up. No recurrence of symptoms was observed at the follow-up, and the mean number of necessary embolization sessions was 1.

### 4.2 Embolic material

We used Onyx for the grouting technique. Onyx is a biocompatible and less adhesive liquid embolic material (1, 16). It can gradually precipitate and solidify upon contact with blood, forming a soft, spongy embolus. Onyx can be delivered to the fistula site, including to the adjacent distal artery and proximal venous drain or pouch, and controlled more precisely than N-butyl cyanoacrylate (1, 16, 20). In high-flow shunts, performing a simple dose injection is impossible. Onyx may require time to migrate through the fistula and into the drainage veins or the involved sinusoids located near the sinus wall. This embolic material is also associated with a lower risk of microcatheter retention than is N-butyl cyanoacrylate. By using Onyx, the microcatheter can be pulled back intermittently from the distal segment to the proximal segment of the sinus, allowing for continual injection and the complete filling of the entire sinus.

If the fistula is not occluded, unintentional distal embolization may occur when the embolic material migrates into the venous systems. Such outcomes may be dangerous and lead to pulmonary embolization (16). Detachable coils can be used to slow the flow within the fistula and provide a physical barrier to other embolic agents (21). Onyx was deposited and polymerized directly at the fistula site and sinus. Therefore, the complete occlusion of the sinus and the fistula can eliminate or minimize the risk of distal embolization and pulmonary embolism.

The cost of using Onyx is less than that of using only conventional coils (for dense packing) (5, 22–25). The nonadhesive property of Onyx eliminates the risk of the microcatheter becoming stuck and allows for prolonged injections into different areas of the sinus, draining vein, and branches of other arterial feeders. This method cannot be realized using the standard coiling technique. The proposed technique can be beneficial in cases where small feeders and twisted vasculature hinder the microcatheter’s distal navigation and adequate coiling (22, 26). Moreover, the technique reduces the economic burden of treatment and shortens the duration of the procedure and, consequently, the exposure to radiation. In our series, the mean numbers of coils used in one patient are 18.55 (11 to 25) with only 1.08 mL (0.3 to 2) Onyx for complete occlusion (Table 1).

Onyx injection into the cavernous sinus may cause lesions or infarctions in the cranial nerves (1, 27, 28) because several cranial nerves are located within or near the cavernous sinus. Nevertheless, in most cases, the combined therapy involves the cavernous sinus and has been indicated to be relatively safe (1). Therefore, the procedure should be performed only in cases where cavernous sinus DAVFs show incomplete occlusion and residual venous reflux after simple coiling. Notably, the proposed grouting technique can be safely applied to other sinuses. No cranial nerve complications or significant consequences were observed in our patients after embolization following the grouting technique.

### 4.3 Limitations

This study had some technical limitations. First of all, the grouting technique was utilized only when the involved sinus or venous varices that contributes nothing or minimally to the normal cerebral outflow. Second, two microcatheters should be buried in the involved sinus at the same time. However, sometimes it was difficult, especially when severe sinus outlet stenosis or occlusion. Finally, this study was limited by its retrospective design and small sample size. A large cohort study may help identify potential differences in clinical outcomes between different groups and subgroups of patients.

## 5 Conclusion

The proposed one stage grouting technique that combines detachable coils and an Onyx injection appears to be promising for the elimination of complex intracranial non-cavernous DAVFs.

## Data availability statement

The raw data supporting the conclusions of this article will be made available by the authors, without undue reservation.

## Ethics statement

The studies involving humans were approved by the Institutional Review Board of Chang Gung Memorial Hospital (IRB No. 202300612B0). The studies were conducted in accordance with the local legislation and institutional requirements. The participants provided their written informed consent to participate in this study. Written informed consent was obtained from the individual(s) for the publication of any potentially identifiable images or data included in this article.

## Author contributions

C-CC: Writing – original draft, Writing – review & editing. C-TC: Conceptualization, Data curation, Investigation, Methodology, Resources, Writing – review & editing. Y-MW: Conceptualization, Methodology, Resources, Supervision, Writing – review & editing. M-CY: Resources, Writing – review & editing. Y-LC: Methodology, Resources, Writing – review & editing. H-FW: Conceptualization, Data curation, Investigation, Methodology, Resources, Supervision, Writing – original draft.
